# MRI features in atypical idiopathic intracranial hypertension

**DOI:** 10.1007/s00330-026-12366-1

**Published:** 2026-02-06

**Authors:** Theresia Knoche, Nehir Guelsoy, Eberhard Siebert, Robin Hollinski, Leon Alexander Danyel

**Affiliations:** 1 Department of Neurology, Charité Universitätsmedizin Berlin—Campus Virchow Klinikum, Berlin, Germany; 2 Berlin Institute of Health at Charité—Universitätsmedizin Berlin, BIH Biomedical Innovation Academy, BIH Charité Clinician Scientist Program, Berlin, Germany; 3 Institute for Neuroradiology, Charité—Universitätsmedizin Berlin, Berlin, Germany

**Keywords:** Idiopathic intracranial hypertension, Magnetic resonance imaging, Visual prognosis, Pseudotumor cerebri

## Abstract

**Objectives:**

Idiopathic intracranial hypertension (IIH) primarily affects obese women of reproductive age. However, IIH can also occur in individuals outside this typical demographic, where it is associated with a more severe clinical course and poorer visual outcome. Characteristic features of IIH have been identified on cerebral MRI but have not been systematically studied in atypical patient subgroups.

**Materials and methods:**

This retrospective cohort study investigated the prevalence of MRI features of IIH across the following subgroups: males, individuals with normal BMI (< 26 kg/m^2^), and patients diagnosed above the age of 45. The presence of empty sella (ES), posterior globe flattening (PGF), optic nerve sheath distension (ONSD), optic nerve tortuosity (ONT), transverse sinus stenosis (TSS), DWI-hyperintensity of the optic nerve head (ONH) and ONH-contrast enhancement were evaluated on MRI. The relationship between MRI features and the visual prognosis was investigated.

**Results:**

The study included 172 patients. ES was most frequent with 87%, followed by ONSD in 60%, TSS in 46%, ONT in 39% and PGF in 37%. ONH-DWI hyperintensity was present in 35%, and ONH-contrast enhancement in 22%. The prevalence of MRI features did not significantly differ across demographic subgroups and between atypical and typical IIH. Regression models did not indicate associations between MRI features and visual outcomes.

**Conclusions:**

MRI features of IIH were equally prevalent in typical and atypical demographics. These findings suggest a consistent radiological presentation of IIH across demographic profiles, indicating a shared imaging phenotype regardless of atypical clinical characteristics. Limitations related to the retrospective design warrant future prospective studies.

**Key Points:**

***Question*** *Do MRI features of IIH differ across demographic subgroups (males, non-obese and older patients) and are they associated with visual outcomes?*

***Findings**** MRI features of IIH were equally prevalent in typical and atypical patients; however, no significant association between imaging findings and visual outcome was observed*.

***Clinical relevance**** While MRI supports the diagnosis of IIH across demographic subgroups, established imaging features do not seem to aid in identifying patients at risk of visual deterioration*.

**Graphical Abstract:**

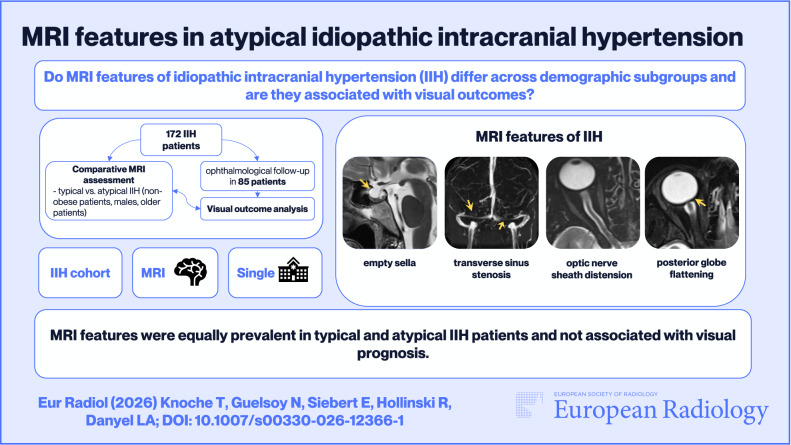

## Introduction

Idiopathic Intracranial Hypertension (IIH) leads to headaches and visual disturbances due to increased intracranial pressure and is strongly associated with elevated BMI. More than 90% of IIH patients are female, and they are typically diagnosed between 25 and 36 years [1–3]. While IIH is considered a rare condition, its incidence has increased up to tenfold over the past 20 years [4].

**Fig. 1:**
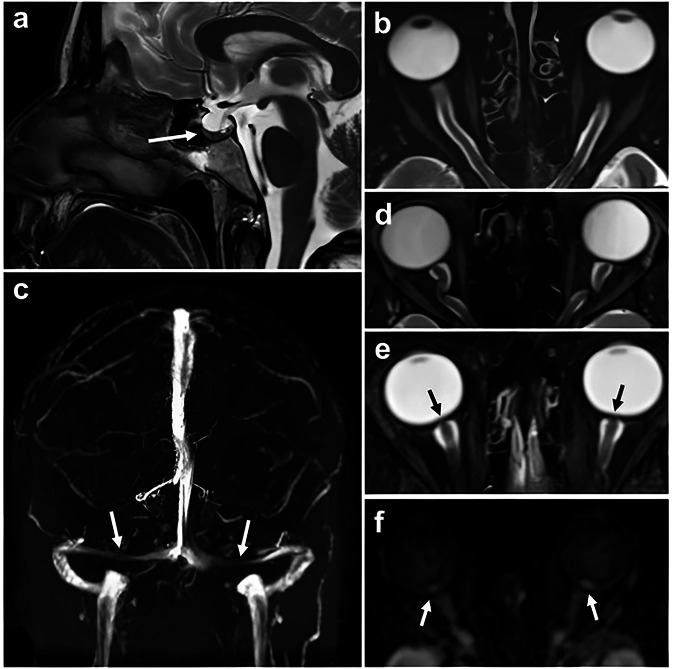
MRI features of IIH. **a** Mid-sagittal T2-weighted MRI shows a partially ES with downward compression of pituitary tissue (white arrow). **b** The axial T2-weighted image reveals a distension of the perioptic subarachnoid space surrounding both optic nerves **c** Time of flight MR venography shows bilateral stenoses of the transverse sinuses (white arrows). **d** Axial T2-weighted MRI demonstrates tortuosity of both optic nerves. **e** Axial T2-weighted MRI depicting bilateral PGF (black arrows). **f** Diffusion weighted imaging (DWI) shows bilateral hyperintensity of the optic nerve head (more prominent on the left side, white arrows)

Neuroimaging is required to exclude secondary causes of increased intracranial pressure [5]. Beside this, several radiographic features are associated with IIH, including the partially empty sella (ES) sign (Fig. 1a), optic nerve sheath distension (ONSD, Fig. 1b), optic nerve tortuosity (ONT, Fig. 1d), posterior globe flattening (PGF, Fig. 1e), and transverse sinus stenosis (TSS, Fig. 1c). Typical MRI features of IIH are presented in Fig. 1. The presence of at least three out of these four findings on MRI is considered highly specific and moderately sensitive for IIH [6, 7]. Recently, a stronger implementation of MRI signs into IIH diagnostic criteria has been proposed [6]. Vision loss, the most serious and irreversible complication of IIH, has prompted investigation of radiographic features for their prognostic relevance to visual outcomes. Yet, current evidence does not support an association between MRI imaging signs and visual prognosis [8].

Certain subpopulations with IIH—such as older individuals, men, and those with normal body weight—have been suggested to be at increased risk of disease progression [9–11]. In these patients, delayed diagnosis and the absence of modifiable risk factors, such as obesity, may contribute to poor visual prognosis. Importantly, the clinical and demographic heterogeneity observed among individuals with IIH may suggest that IIH represents a spectrum of related but distinct disease entities.

However, individuals who fall outside the typical demographic profile of IIH patients have not been individually characterised on neuroimaging before. As such, a potential association between MRI findings and visual prognosis in atypical IIH has not yet been systematically investigated. We suspected that the prevalence and distribution of MRI features vary across IIH subgroups, possibly affecting the diagnostic validity of established MRI criteria. To this end, we analysed a large cohort of IIH patients and compared the MRI features of typical IIH patients—defined as women under the age of 45 with elevated BMI—with those of atypical demographic subgroups, including men, individuals diagnosed at an older age, and those with normal BMI. Finally, we aimed to evaluate the predictive value of MRI signs in relation to visual outcomes.

## Materials and methods

This retrospective cohort study was approved by our institutional ethics committee and conducted in accordance with the Declaration of Helsinki. The need for informed consent was waived for retrospective data extraction and analysis by our institutional review board. This study complies with the reporting guidelines within the Strengthening the Reporting of Observational Studies in Epidemiology (STROBE) Statement.

All patients were treated at Charité—Universitätsmedizin Berlin, which comprises three tertiary care university hospitals. Database enquiry identified all patients treated under the definite or suspected diagnosis of IIH (ICD-10 code: G93.2) between January 2004 and October 2020. Our IIH cohort has been described in detail elsewhere [12]. Briefly summarised, the acquired patient datasets included demographic and disease-specific parameters upon presentation and follow-up, as well as therapeutic interventions. Each patient had CSF analysis with documentation of CSF opening pressure, cerebral MRI, neuro-ophthalmological and neurological examination to exclude alternative causes of optic disc oedema or intracranial hypertension. All patients were treated according to best practice with recommendations of weight loss for overweight or obese patients, pharmacological treatment with acetazolamide, topiramate, and/or furosemide, as well as invasive treatment options such as a ventriculoperitoneal shunt or bariatric surgery, if indicated. This study included all patients with a confirmed diagnosis of IIH or IIH without papilledema (IIH-WOP) according to the IIH diagnostic criteria by Friedman et al [5], provided that the initial diagnostic MRI studies were available. For the comparative assessment of IIH MRI features, three patient subgroups of atypical IIH were defined: (1) male patients, (2) non-obese patients (BMI < 26 kg/m^2^), and (3) patients aged 45 years or older at the time of IIH diagnosis [13]. Atypical groups were not compared to each other due to the overlap of individual patients between atypical groups.

MRI scans were acquired using either 1.5 or 3-Tesla scanners. As an MRI was performed during routine diagnostic work-up at the time of diagnosis, imaging protocols varied slightly. Availability of T1- and T2-weighted sequences in two orthogonal planes to exclude structural brain lesions was a prerequisite for study inclusion, as well as either a non-contrast MR-venography or a post-gadolinium T1-weighted sequence to rule out cerebral venous sinus thrombosis in all atypical patients. If the archived MRI scans did not meet the prerequisite, corresponding patient cases were excluded from the analyses. A board-certified senior neuroradiologist (R.H.), who was blinded to clinical patient data and the study group assignment, performed the MRI assessment. Maximum pituitary gland height was measured on midsagittal T1- or T2-weighted images [14]. The presence of partial ES was defined based on the extent of suprasellar herniation of CSF, using a threshold of moderate herniation defined as ≥ 1/3 of the sella height [15, 16]. Presence of ONSD was defined as either (1) uni- or bilateral perioptic subarachnoid space > 2 mm in the coronal plane on T2-weighted images [6, 17, 18] or (2) an optic nerve sheath (ONS) diameter exceeding > 5.8 mm in the axial imaging plane perpendicular to the axis of the optic nerve, measured 8 mm posterior to the posterior circumference of the globe [14, 19]. ONT and PGF were assessed qualitatively on axial T2-weighted images. The presence of optic nerve head DWI-hyperintensity (ONH-DWIH) was qualitatively evaluated on diffusion weighted imaging (DWI), if available. Similarly, the presence of optic nerve head contrast enhancement (ONH-CE) was qualitatively evaluated on post-gadolinium weighted imaging, if conducted. Transverse sinus stenoses (TSS) were evaluated by a method originally published by Carvalho et al, which has been modified and adapted in more recent studies [6, 8, 20]. Stenoses were graded 1–4 on each side: 1. 33% stenosis, 2. 33–66% stenosis, 3. 66% stenosis and 4. hypoplasia or agenesia. A score (index of transverse sinus stenosis [ITSS]) was calculated by multiplying the two grades, and a value of ≥4 was considered indicative of IIH. MRI scans that did not permit a reliable assessment of a specific imaging feature were excluded from the analysis for that same feature.

Visual outcomes were recorded, if a neuro-ophthalmologic follow-up examination at least 6 months after initial diagnosis was available. Best corrected visual acuity (BCVA) of the most severely affected eye was transformed to logMAR (logarithm of the minimum angle of resolution) for statistical analysis (logMAR = −log (decimal visual acuity)). Visual field perimetric mean deviation (MD) in decibel [dB] Octopus 900 perimeter (Haag-Streit) using the 30-2° tendency-oriented perimetry. Visual field perimetric MD of > 6.0 dB in at least one eye was considered abnormal. Funduscopic papilledema grading on the modified Frisén scale of the most severely affected eye (i.e. the higher value) was extracted from ophthalmologic reports [21]. The presence of optic nerve atrophy was noted.

For regression analysis, the visual outcome was dichotomously defined as either the absence or the presence of poor visual outcome in at least one eye. Poor visual outcome was defined as follows:

- Visual acuity ≥ 0.2 logMAR and/or perimetric MD of > 6.0 dB
- Decline of visual acuity by ≥ 0.2 logMAR and/or worsening of papilledema by one category and/or new occurrence of optic disc atrophy

### Statistical analysis

Statistical analyses were performed using IBM SPSS Statistics software (IBM SPSS Statistics, Version 30.0) and GraphPad Prism (Version 8.0.0 for Windows, GraphPad Software). Categorical variables were expressed in frequencies and percentages, continuous normally distributed variables as mean and standard deviation and non-parametric variables as median and interquartile range (IQR), as appropriate. Missing data were handled by pairwise deletion and reporting of valid percentages. Q-Q plots and histograms were used to test for the normal distribution of continuous variables. Univariate group comparisons were computed using the chi-square test for categorical and two-sided *t*-test for continuous variables. Spearman’s rank correlation test was used for univariable associations. Forward stepwise binary logistic regression was conducted to test the association of MRI signs with the visual outcome, using poor visual outcome as the dependent variable. Each MRI sign was defined as a dichotomous independent variable comparing the absence and presence of ES, ONSD, PGF, and TSS using absence as the reference category. Univariable regression was applied to test the association between the presence and absence of a number (≥ 2, ≥ 3 and 4) of MRI signs and the visual outcome. For regression analyses, listwise deletion was applied, excluding cases with missing values for any of the included MRI signs. Results of the regression analysis are presented as odds ratios (OR) and 95% confidence intervals (95% CI). Goodness-of-fit was assessed using the Hosmer-Lemeshow-test. The level of significance was set at a two-sided *p*-value < 0.05.

## Results

**Fig. 2:**
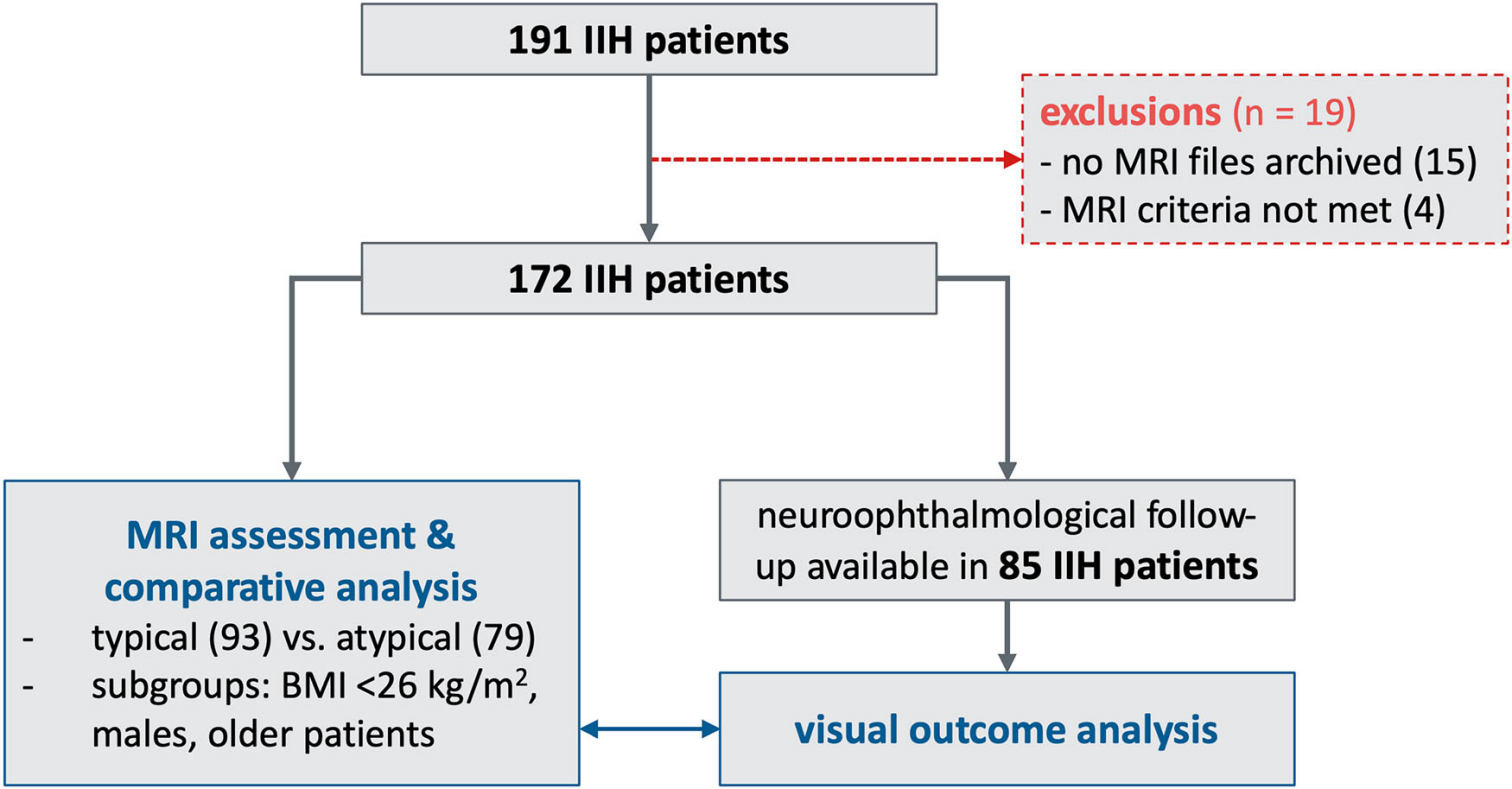
Flow chart of the inclusion/exclusion process. BMI, body-mass-index; IIH, idiopathic intracranial hypertension

Of 191 pre-identified IIH/IIHWOP-patients, 19 did not fulfil the minimum MRI protocol criteria, resulting in a final study cohort of 172 patients (85% female). Mean age at diagnosis was 35 ± 12.5 years (range: 14–75). Seven patients (4%) were diagnosed with IIHWOP. The inclusion/exclusion process is presented in Fig. 2.

Patients with atypical demographic characteristics accounted for 46% of the study cohort (*n* = 79), including (1) male patients (*n* = 26, 15%), (2) older patients (≥ 45 years at the time of IIH diagnosis, *n* = 39, 23%), and (3) patients with BMI < 26 kg/m^2^ (*n* = 29, 17%). Male patients were significantly older than females (m: 42.1 ± 15.4 vs f: 34.2 ± 11.6 years, *p* = 0.001) and had lower BMI than the typical IIH cohort (*p* = 0.01, see Table 1). Table 1 summarizes core demographic and clinical data of the IIH study cohort.

**Table 1:** Baseline cohort characteristics

	All patients	Typical IIH	Atypical IIH patients, *n* = 79		
	*n* = 172	*n* = 93	Males *n* = 26	Older (≥ 45 y) *n* = 39	BMI < 26 kg/m^2^ *n* = 29
Age	35.5 ± 12.5	29.2 ± 7.6	42.1 ± 15.4	53.7 ± 8.0	33.3 ± 9.8
BMI [kg/m^2^]	33.6 ± 7.8	35.8 ± 6.8	31.7 ± 8.9	33.7 ± 7.0	23.7 ± 1.6
CSF-OP [cmH_2_O]	35.9 ± 8.4	37.4 ± 8.3	35.8 ± 9.9	34.4 ± 7.2	32.3 ± 6.7
Visual acuity, first visit [logMAR]	0.1 ± 0.20	0.1 ± 0.2	0.17 ± 0.2	0.08 ± 0.16	0.14 ± 0.18
Visual field MD, first visit [dB]	6.0 ± 6.3	8.0 ± 7.4	5.7 ± 6.1	3.9 ± 3.8	2.3 ± 1.5
Frisen score, first visit (median + IQR)	2.0 (3–1)	2 (3–1)	3 (3–1)	2 (3–2)	2 (3–1)

*BMI* body-mass-index, *CSF-OP* cerebrospinal fluid opening pressure, *MD* mean deviation, *dB* decibel, *IQR* inter-quartile range

### Frequency of imaging signs

Among the 4 imaging features that are a part of IIH diagnostic criteria proposed by Friedman et al (i.e. ES, ONSD, PGF, TSS), at least one MRI feature was found in 94.0% and ≥ 3 imaging signs in 39.4% of all patients. All 4 MRI features were found in 9.7% of patients.

**Fig. 3:**
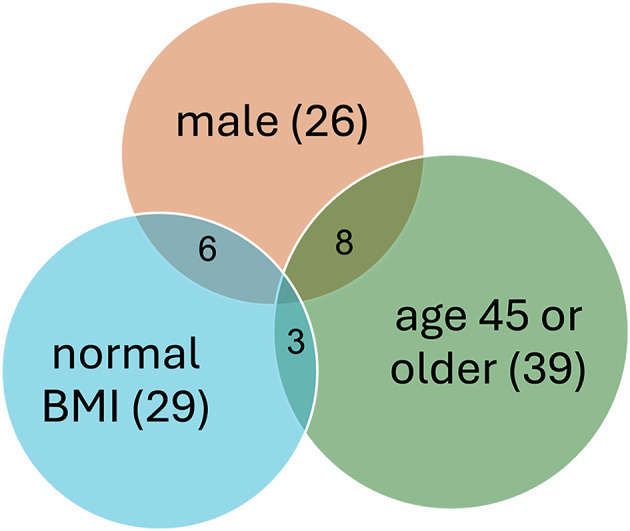
“Venn diagram” of the atypical patient cohort (*n* = 79), indicating partial overlap between subgroups

**Fig. 4:**
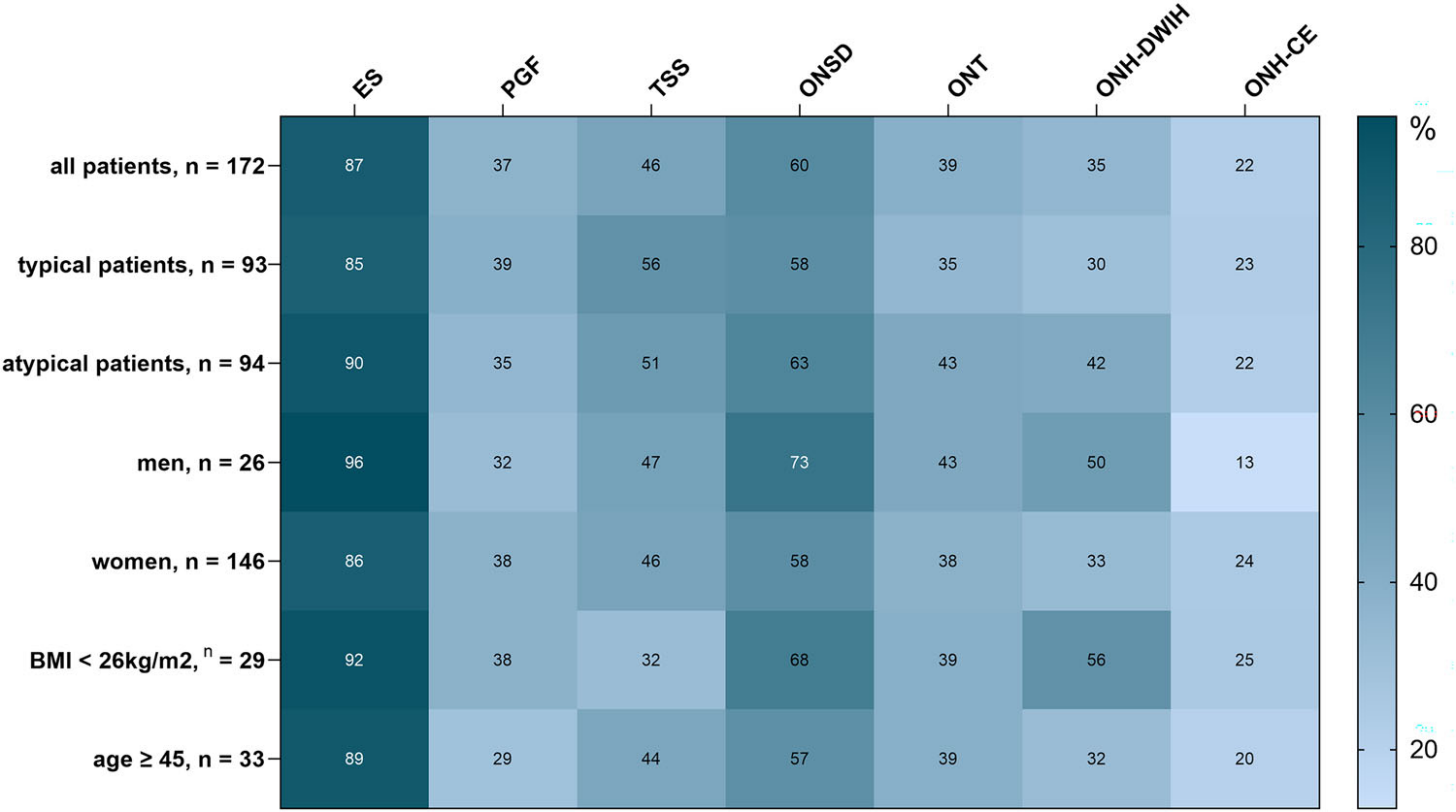
Heatmap illustrating the prevalence of MRI features across different subgroups of the IIH cohort (given in percentages for each subgroup). Corresponding data in supplemental Table 1. ONT, optic nerve tortuosity; PGF, posterior globe flattening; ONSD, optic nerve sheath distension; ES, empty sella; TSS, transverse sinus stenosis; ONH-DWIH, optic nerve head diffusion weighted imaging hyperintensity; ONH-CE, optic nerve head contrast enhancement

ES was present in 87.2% of cases, ONSD in 60.3%, ONT in 38.9%, PGF in 37.1%, and TSS in 46%. Of 97 patients with available DWI, 35.1% had ONH-DWI hyperintensity. Of 104 patients, who had received post gadolinium imaging, 22.1% displayed ONH-contrast enhancement. The prevalence of MRI features did not significantly differ between males and females. ONSD was more frequent in males than in females, but not statistically significant (m: 73%, f: 58%, *p* = 0.20). The frequency of MRI features was comparable between patients with a normal body mass index (BMI) and those with a BMI ≥ 26 kg/m², as well as between patients diagnosed at ≥ 45 years of age and those diagnosed before 45 years. Furthermore, neither the presence of individual MRI signs nor the number of signs observed (i.e. 1, 2, 3, or 4) differed significantly between typical and atypical patient groups. Frequencies of the individual MRI signs in relation to patient subgroups are visualised in Figs. 3 and 4 (corresponding data are presented in supplemental Table 1).

Maximum pituitary gland height showed a negative correlation with age (correlation coefficient *r* = −0.42, *p* < 0.001). However, the prevalence of at least moderate suprasellar herniation (defined by Yuh et al as a threshold of ≥ 1/3 of the sella height) was similarly frequent between the group of older and the group of younger patients (87% vs 89%, *p* = 0.68). No associations were found between ITSS or pituitary gland height and CSF opening pressure.

MRI studies were performed using a field strength of 1.5-Tesla in 84% and 3.0-Tesla in 16% of cases. The prevalence of MRI features did not significantly differ between 1.5-Tesla and 3-Tesla scanners.

### Visual outcomes

Visual follow-up data at a minimum of 6 months after initial diagnosis were available in a subset of 85 patients (49%, 85/172), who were consequently included in the visual outcome analysis. Neither age, BMI nor CSF opening pressure was associated with poor visual outcome. The number of MRI signs did not significantly differ between the groups of patients with and without poor visual outcome (2.1 vs 2.3, *p* = 0.56). Univariable analyses did not show an association between any single MRI sign (ES, ONSD, PGF, TSS, ONT), nor the number of detected MRI signs (≥ 2, ≥ 3 or = 4) and the visual outcome. A multivariable regression model including ES, ONSD, PGF, and TSS as independent variables was not associated with an increased likelihood of poor visual outcome. Results of the regression analysis are summarised in Table 2.

**Table 2:** Regression analysis regarding visual outcome

	Univariable		Multivariable	
	OR (95% CI)^1^	*p*-value	OR (95% CI)^2^	*p*-value
Age	1.02 (0.98–1.06)	0.42	-	-
Sex	1.85 (0.51–6.68)	0.35	-	-
BMI	1.01 (0.96–1.07)	0.64	-	-
CSF opening pressure	1.02 (0.96–1.07)	0.52	-	-
Disease duration	1.08 (0.97–1.21)	0.14	-	-
ES	1.43 (0.35–5.84)	0.62	0.83 (0.14–5.11)	0.85
ONSD	1.68 (0.63–4.51)	0.30	2.18 (0.60–7.96)	0.24
PGF	1.67 (0.65–4.31)	0.29	0.96 (0.28–3.34)	0.95
TSS	0.78 (0.26–2.31)	0.65	0.72 (0.19–2.68)	0.62
ONT	0.79 (0.31–2.03)	0.62	**-**	**-**
≥ 2 MRI signs	1.23 (0.41–3.65)	0.72	**-**	**-**
≥ 3 MRI signs	1.86 (0.72–4.77)	0.20	**-**	**-**
4 MRI signs	0.64 (0.12–3.41)	0.60	**-**	**-**

*CSF* cerebrospinal fluid, *OR* odds ratio, *95% CI* 95% confidence interval

^1^ Obtained by univariable binary regression

^2^ Obtained by multivariable stepwise forward regression (Nagelkerke R-square: 0.05; Hosmer-Lemeshow test: *p* = 0.31)

Patients who were lost to follow-up were older than patients in the follow-up cohort (*p* = 0.05, 37 ± 13 vs 34 ± 11 years). Other characteristics (sex, BMI, CSF opening pressure, visual acuity, Frisén score) of patients lost to follow-up did not significantly differ from those with follow-up (Supplemental Table 2).

## Discussion

This study represents the largest investigation of MRI features in atypical IIH patients to date, incorporating data collected over 16 years from three tertiary care centres. The frequency of IIH MRI features did not significantly differ between typical and atypical demographic subgroups, including males, non-obese patients and patients diagnosed at an older age. Furthermore, MRI signs were not associated with visual prognosis.

ES was the most frequently observed imaging sign (87%), higher than rates reported in recent studies, which typically range from 65% to 80% when applying similar radiological criteria [16, 22, 23]. This higher prevalence may be related to the slightly older age distribution of patients in the present cohort, as ES has been reported to occur more frequently in older adults with IIH compared to younger patients [24]. Consistent with prior observations, pituitary gland height in our cohort was inversely correlated with age [25, 26]. However, the proportion of older patients exhibiting ES—defined as suprasellar herniation exceeding one-third of the sella height, was comparable to that of younger patients. This finding suggests that the age-related decline in pituitary height does not explain the rate of ES in older IIH patients and supports its diagnostic utility across all age groups when the definition by Yuh et al is applied [15]. While our criterion for ES represents a pragmatic radiological convention, rather than a pathological threshold; therefore, ES should be interpreted in conjunction with the clinical presentation and other imaging findings [26].

Although ONSD appeared more common in male patients, the difference did not reach statistical significance. Overall, we found no differences in the distribution of individual MRI features between IIH subgroups. Similarly, the cumulative number of MRI signs per individual did not differ between groups. These findings imply that despite differences in clinical presentation and outcome, the radiological phenotype of IIH remains consistent across demographic subgroups. This may be explained by the fact that these imaging findings reflect a shared pathophysiological mechanism, such as sustained elevation of intracranial pressure, that is independent of demographic factors. As described before, pituitary gland height was inversely correlated with age [25]. However, the proportion of older patients with significant suprasellar herniation was comparable to that of younger patients. This finding suggests that the age-related decline in gland height does not account for the high rate of ES in older IIH patients and supports its utility as a diagnostic sign across all age groups. Neither the presence of individual MRI signs nor their cumulative presence was associated with poor visual outcome, which is consistent with the results of prior investigations [8, 10]. Furthermore, other variables that are traditionally considered to be risk factors, including age, BMI, and CSF opening pressure, were not predictive of visual deterioration in this cohort [12]. Visual outcomes in IIH may be more dependent upon individual factors such as optic nerve resilience, timely diagnosis, and responsiveness to treatment, which are not adequately captured by MRI findings. Our findings support the validity of MRI criteria as a complementary diagnostic tool to improve recognition of IIH across all patient subgroups, while acknowledging that these imaging signs likely represent secondary correlates of raised intracranial pressure rather than predictors of disease severity or outcome.

The lack of predictive value of MRI in IIH emphasises the need for more sensitive biomarkers of disease progression. While the current imaging criteria support the diagnostic process, their utility for risk stratification appears limited. This may be particularly relevant in atypical IIH subgroups, who are underrepresented in clinical studies but may show different courses of the disease. Although our study did not find radiographic differences between subgroups, the deviating clinical course and treatment response in certain subsets of IIH patients warrant further investigation.

Moreover, our data question the current paradigm that clusters IIH patients solely by demographic criteria. The overlapping neuroimaging profiles among patients suggest that pathophysiological mechanisms may converge despite differences in sex, age, or BMI. Nonetheless, it remains possible that subtle differences in MRI features or clinical presentation, not captured in this study, exist between subgroups and could inform more individualised treatment strategies.

This study has limitations that warrant cautious interpretation of its findings. First, while MRI interpretation was conducted by an experienced neuroradiologist blinded to clinical data, variability in imaging protocols may have affected the detection of some features. However, we found no significant differences in the frequency of MRI signs between 1.5-Tesla and 3.0-Tesla scans, suggesting that field strength did not substantially impact feature detection. Second, although recent studies have demonstrated moderate to excellent interrater agreement for IIH-associated MRI findings [27], some reader-dependent variability must be expected in our analyses, which were conducted by one senior neuroradiologist. Third, follow-up data for visual outcomes were only available for a subset of patients. Reasons for missing visual follow-up primarily included loss to follow-up (most likely due to transfer to external care providers or improvement/remission of symptoms) and lack of ophthalmologic documentation. This likely reduced the statistical power to identify subtle prognostic associations between MRI features and long-term visual outcomes. Lastly, the retrospective nature of our study introduces the possibility of selection bias, as patients would more likely be diagnosed with IIH, if they exhibited radiographic features of the disease. Nevertheless, most patients of our study cohort were diagnosed with definitive IIH according to Friedman criteria, which base IIH diagnosis on the presence of papilledema and elevated CSF opening pressure and do not require the presence of radiographic features of IIH.

## Conclusion

In this study, MRI features commonly associated with IIH were observed across both typical and atypical subgroups, including males, older individuals, and patients with a normal BMI. In line with prior investigations, no association between the presence or number of MRI features and visual outcome was observed. The validity of our prevalence estimates, subgroup comparisons and regression analysis is limited by the retrospective nature of our study, with varying imaging protocols and a proportion of patients missing visual follow-up. However, our findings indicate that the radiological presentation of IIH may largely be consistent across demographic profiles, supporting the concept of a shared imaging phenotype, thereby strengthening the role of MRI in the diagnosis of IIH. Given the limitations, multicentre, prospective investigations with standardised MRI protocols and systematic ophthalmologic follow-up are required to confirm these observations and to clarify the role of imaging biomarkers in risk stratification of IIH.

## Supplementary information

ELECTRONIC SUPPLEMENTARY MATERIAL

## Data Availability

The datasets generated for the current study are not publicly available but are available from the corresponding author on reasonable request by qualified researchers.

## Abbreviations

BCVA: Best corrected visual acuity
BMI: Body-mass-index
CSF: Cerebrospinal fluid
DWI: Diffusion weighted imaging
ES: Empty sella
IIH: Idiopathic intracranial hypertension
IIH-WOP: Idiopathic intracranial hypertension without papilledema
IQR: Interquartile range
ITSS: Index of transverse sinus stenosis
logMAR: logarithm of the minimum angle of resolution
MD: Mean deviation
ONH: Optic nerve head
ONH-CE: Optic nerve head contrast enhancement
ONH-DWIH: Optic nerve head diffusion weighted imaging hyperintensity
ONS: Optic nerve sheath
ONSD: Optic nerve sheath distension
ONT: Optic nerve tortuosity
OR: Odds-ratio
PGF: Posterior globe flattening
TSS: Transverse sinus stenosis
