# Supplementary material for: MRI features in atypical idiopathic intracranial hypertension
Source: Eur Radiol. 2026 Feb 6;36(7):5432–41. doi: 10.1007/s00330-026-12366-1 (PMC13282342; doi:10.1007/s00330-026-12366-1)
Supplement: Supplementary file 1 — ELECTRONIC SUPPLEMENTARY MATERIAL [file 330_2026_12366_MOESM1_ESM.pdf]

MRI features in atypical Idiopathic Intracranial Hypertension

ELECTRONIC SUPPLEMENTARY MATERIAL

Supplemental Table 1

Frequencies of MRI features in the study cohort and with regard to subgroups.

	empty sella	posterior globe flattening % (n)	transverse sinus stenosis	optic nerve sheath distension	optic nerve tortuosity	optic nerve head – DWI hyperintensity	optic nerve head enhancement
all patients, n=172	87% (136/156)	37% (62/167)	46% (54/117)	60% (91/151)	39% (62/159)	35% (34/97)	22% (23/104)
males, n=26	95% (21/22)	32% (8/25)	47% (7/15)	73% (16/22)	43% (10/23)	50% (5/10)	13% (2/16)
women, n=146	86% (115/134)	38% (54/142)	46% (47/102)	58% (75/129)	38% (52/136)	33% (29/87)	24% (21/88)
normal BMI (BMI <26kg/m^2), n=29	92% (24/26)	38% (11/29)	32% (6/19)	68% (19/28)	39% (12/27)	56% (9/16)	25% (5/20)
BMI >/= 26 kg/m^2, n=141	86% (110/128)	38% (51/136)	49% (47/96)	59% (71/121)	38% (49/130)	30% (24/80)	22% (18/82)
age 45 or **older**, n =39	89% (33/37)	29% (11/38)	44% (10/23)	57% (21/37)	39% (12/38)	32% (8/25)	20% (5/25)
age 40 or **older**, n=59	91% (48/53)	31% (18/58)	54% (21/39)	63% (35/56)	39% (19/58)	35% (14/40)	21% (8/39)
age <40 years, n=113	85% (88/103)	40% (44/109)	42% (33/78)	59% (56/95)	43% (43/101)	35% (20/57)	23% (15/65)
atypical patients, n=93	90% (64/71)	35% (27/77)	51% (25/49)	63% (45/72)	43% (32/74)	42% (18/43)	22% (11/51)
typical patients, n=93	85% (72/85)	39% (35/90)	56% (38/68)	58% (46/79)	35% (30/85)	30% (16/54)	23% (12/53)

Supplemental Table 2. Characteristics of the follow-up cohort and patients lost to follow-up.

	follow-up cohort (n = 85)	patients lost to follow-up (n = 87)	p-value
**age**	33.5 ± 11.2	37.3 ± 13.4	0.05[1]
**female/male**	f = 74; m = 11	f = 72; m = 15	0.4[2]
**BMI [kg/m^2]**	33.7 ± 7.9	33.4 ± 7.7	0.8[1]
**CSF-OP [cmH_2O]**	35.9 ± 8.4	36.4 ± 8.0	0.7[1]
**Frisén grade[3] (median & IQR)**	2 (2)	2 (1)	0.4[4]
**BCVA [logMAR]**	0.16 ± 0.40	0.17 ± 0.39	0.9[1]

[1]two-sided p-values calculated by independent samples t-test.
[2]calculated by Fisher's exact test.
[3]available in 133 patients.
[4]calculated by Mann-Whitney-U-test.
